# Multiple aggregated yellow-white globules: a basal cell carcinoma dermoscopic feature in skin of color

**DOI:** 10.11604/pamj.2024.48.101.44152

**Published:** 2024-07-12

**Authors:** Sara Oulad Ali, Jihane Belcadi, Lina Benchekroun, Sara El Ghaffouli, Samia El Hilali, Karima Senouci, Mariame Meziane

**Affiliations:** 1 Department of Dermatology, Mohammed V University in Rabat, Ibn Sina University Hospital, Rabat, Morocco,; 2 Department of Histopathology, Mohammed V University in Rabat, Ibn Sina University Hospital, Rabat, Morocco,; 3 Community Medicine Laboratory (Public Health, Preventive Medicine, Hygiene), Mohammed V University in Rabat, Ibn Sina University Hospital, Rabat, Morocco

**Keywords:** Dermoscopy, multiple aggregated yellow-white globules, basal cell carcinoma

## To the editors of the Pan African Medical Journal

The dermoscopic structures known as multiple aggregated yellow-white (MAY) globules were initially described as highly specific for high-risk non-pigmented basal cell carcinoma (BCC) [1]. However, recent studies revealed that they are a significant dermoscopic criterion for diagnosing BCC, regardless of histological subtype or pigmentation [2,3]. While BCC is the most common malignant skin tumor, the pigmented form represents less than 10% of BCC cases [4]. Therefore, the focus of this study was to elucidate the dermoscopic features of pigmented BCCs in Moroccan patients, particularly MAY globules, and to correlate them with the degree of pigmentation and the BCC subtype.

**Figure 1:**
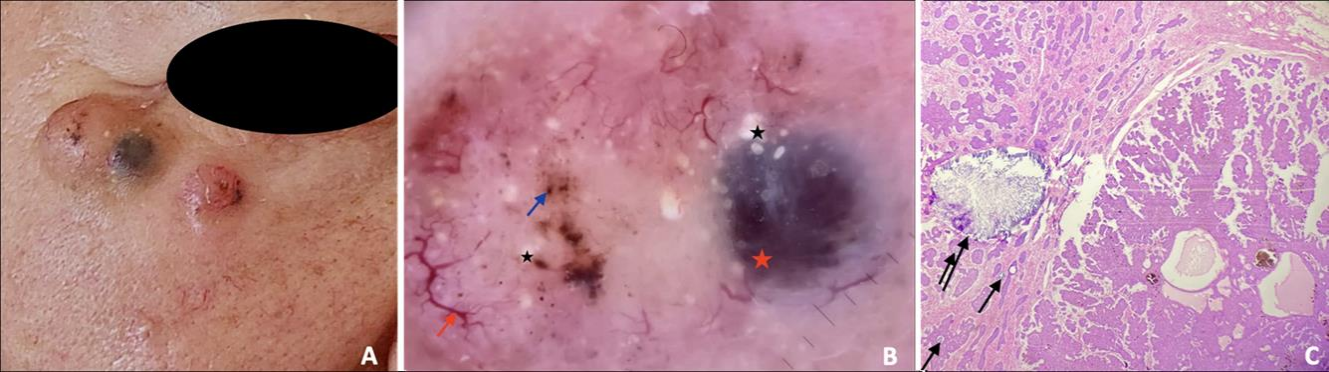
representation of MAY globules in a pigmented nodular BCC: A) two contiguous nodular BCCs in a 64-year-old patient; B) dermoscopic of two contiguous nodular BCC showing MAY globules (black asterisks), a large gray-blue ovoid nest (red asterisk), arborizing vessels (red arrow), and brown dots (blue arrow); C) histopathologic image showing the presence of multiple calcifications (black arrows) in a pigmented basal cell carcinoma

A prospective monocentric study was realized at the Dermatology Department of the Ibn Sina University Hospital in Rabat, Morocco for 24 months. Clinical and dermoscopic images of histopathologically confirmed BCCs were evaluated. One hundred and twenty (120) BCCs in 92 patients were included with a mean age and SD of 59 years ± 14.55. Fifty-seven (57) patients were men and 35 were women. The predominant skin phototype was phototype IV, found in 39 patients. Our series included 76 nodular BCCs (63%), 35 superficial BCCs (29%), 7 infiltrating BCCs (6%) and 2 morpheaform BCCs (2%). All the BCCs included in this study were pigmented, and 79 (65.8%) were heavily pigmented with a pigmentation involving more than 75% of the lesion. Forty-three (35.8%) were located in the nasofrontal zone and 34 (28.3%) in the peri-orbital area. The most frequent dermoscopic criterion was arborizing vessels found in 95 of the cases (79.2%), followed by grey-blue ovoid nests, ulcerations/erosions, and shiny white structures found in 63.3%, 57.6%, and 33.3% of the cases respectively. MAY globules were found in 11 BCCs (9.2%). They were present in 12% of the nodular BCCs (n = 9) (Figure 1A, B) and in 5.9% of the superficial subtype (n = 2). Furthermore, we did not find this dermoscopic criterion in infiltrating or morpheaform subtypes and no association was noted with any specific anatomical site or degree of pigmentation.

The histopathological examination of BCCs with MAY Globules from our series reveals calcifications within the tumor proliferation (Figure 1C) Multiple aggregated yellow-white globules were firstly described as a new BCC dermoscopic criterion that was negatively associated with superficial BCC and positively associated with deeper-seated, histologic, higher-grade tumor subtypes [1]. In our study, this dermoscopic feature was only found in low-risk BCC subtypes (superficial and nodular) and was not observed in other high-risk subtypes (infiltrating and morpheaform). Multiple aggregated yellow-white globules are different from milia-like cysts; as they look bright under polarized dermoscopy unlike milia-like cysts, which only take on this aspect under non-polarized light [5]. Contrary to milia-like cysts that correspond to keratin-filled cysts histologically, MAY globules are correlated with intratumoral calcifications [6]. In 2022, a multicentric Indian study including 143 BCCs in dark-skinned patients found that MAY globules were present in 14% of the pigmented BCCs, without being correlated to any specific histological subtype [3].

In a recent publication, the authors assessed the diagnostic accuracy of MAY globules in a retrospective case-control study involving pigmented and non-pigmented basal cell carcinomas (BCCs) of various histological types. Control cases, randomly selected from a database, comprised both benign and malignant tumors. A total of 389 BCCs were analyzed, with MAY globules detected in 192 (49%) cases within the BCC group compared to only 25 cases (6.4%) in the control group. The presence of MAY globules was notably significant across three histological subtypes, including superficial BCCs, underscoring their importance as a major dermoscopic criterion for diagnosing BCCs, regardless of histological subtype or pigmentation status [2]. Our results were consistent with these two recent studies.

## Conclusion

Through this study, we can confirm that MAY globules are also a dermoscopic feature in pigmented BCCs among patients in a dark-skinned population, and in low-risk BCC subtypes not only in high-risk subtypes as it was first described.
